# Hemophagocytic Lymphohistiocytosis Following COVID-19 Infection

**DOI:** 10.7759/cureus.34307

**Published:** 2023-01-28

**Authors:** Guarina Molina, Rafael Contreras, Kyle Coombes, Thilini Walgamage, Maria A Perozo, Martha T DesBiens

**Affiliations:** 1 Internal Medicine, Danbury Hospital, Danbury, USA; 2 School of Medicine, American University of the Caribbean, Cupecoy, SXM; 3 Infectious Disease, Danbury Hospital, Danbury, USA

**Keywords:** disseminated intravascular coagulation (dic), soluble interleukin receptors, inflammatory mediators, pancytopenia, hemophagocytic lymphohistiocytosis (hlh), covid 19

## Abstract

Coronavirus disease 2019 (COVID-19), caused by severe acute respiratory syndrome coronavirus 2 (SARS-CoV-2), has been associated with multiple inflammatory symptoms involving several organ systems, including hematologic manifestations. Hemophagocytic lymphohistiocytosis (HLH) is a life-threatening syndrome caused by excessive inflammation in the absence of immune regulation. We present the case of a patient with HLH secondary to dysregulated inflammatory response following COVID-19; we also describe the diagnostic and management challenges associated with the condition.

## Introduction

Coronavirus disease 2019 (COVID-19), caused by severe acute respiratory syndrome coronavirus 2 (SARS-CoV-2), results in diseases of variable severity in different individuals. Recent advances in our understanding of SARS-CoV-2 infection suggest that viral activation of macrophages may form inflammasomes, which work to control viral infection, drive inflammatory cell death, and release potent inflammatory mediators [1,2]. In susceptible individuals, this process may cause diverse potential inflammatory pathology and clinical manifestations.

Hemophagocytic lymphohistiocytosis (HLH) is a rare life-threatening syndrome that can occur as a secondary illness triggered by viral infection [1]. Under physiologic conditions, macrophage activation in response to infection is limited by immune feedback regulation. However, without appropriate feedback regulation, macrophage activation continues, leading to excessive pro-inflammatory cytokines and subsequent tissue damage. This phenomenon may lead to a spectrum of hyper-inflammatory illnesses, including macrophage activation syndrome (MAS), adult-onset Still’s disease, multi-system inflammatory syndrome (MIS), and secondary HLH (sHLH) [2-6].

Cases of HLH have been reported in patients with COVID-19 and may relate to post-infectious inflammatory sequelae [7,8]. Here we present a case of HLH that developed following COVID-19 pneumonia. We also engage in a review of the relevant literature.

## Case presentation

A 73-year-old man without any chronic medical conditions developed PCR-positive COVID-19 pneumonia in December 2020, for which he did not require hospitalization. In April 2021, after four months of incomplete improvement, his health declined further, characterized by chronically recurrent fever, headache, arthralgia, and malaise. He did not seek medical attention until September 2021, when he presented to another institution with fever, confusion, and escalating shock with disseminated intravascular coagulopathy (DIC). Infectious evaluation including multiple cultures was unremarkable. His shock and DIC progressed, requiring escalation of care and eventual transfer to our institution.

On admission, his temperature was 39.7 °C; he had a heart rate of 125 bpm and blood pressure of 108/68 mmHg. A thorough physical exam was unremarkable, aside from short-term memory deficits without meningeal signs.

Initial blood work is summarized in Table 1, including workup for infectious (Table 2), rheumatologic, and hematologic causes (Table 3).

**Table 1:** Initial blood work WBC: white blood cell count; AST: aspartate aminotransferase; ALT: alanine aminotransferase

Variables	Values
White blood cell (WBC) count	5.5 K/uL
Hemoglobin	7.8 g/dL
Platelet count	13,000 x 10^9^/L
Procalcitonin	3.08 ng/mL (Ref. <0.1)
Ferritin	60,435 ng/mL (Ref. 20-250 ng/mL)
Triglycerides	268 mg/dL (Ref. <149)
ALT	182 U/L
AST	153 U/L

**Table 2:** Serologies and NAATs *Hbs antibody: 7.5. **Positive Ab suggestive of past exposure to CMV infection. ***Overall test considered unremarkable, as the patient had no prior TB exposure, and the indeterminate result was thought to be due to concomitant lymphopenia HIV: human immunodeficiency virus; Ag: antigen; Ab: antibody; COVID-19: coronavirus disease 2019; PCR: polymerase chain reaction; RSV: respiratory syncytial virus; RPR: rapid plasma reagin; IgG: immunoglobulin G; IgM: immunoglobulin M; CMV: cytomegalovirus; TB: tuberculosis; HAV: hepatitis virus A; HBV: hepatitis virus B; HCV: hepatitis virus C

Tests	Results
HIV Ag and Ab screen	Non-reactive
COVID-19 PCR	Negative
Influenza (A and B) PCR	Negative
RSV PCR	Negative
Hepatitis A, B, and C panel	HAV non-reactive, HBVsAb reactive*, HBVAg non-reactive, HBVcAb non-reactive, HCV non-reactive
RPR for syphilis	Negative
Bartonella henselae IgG/IgM	<1:64/<1:16
Bartonella quintana IgG/IgM	<1:64/<1:16
Babesia PCR	Not detected
Anaplasma PCR	Not detected
Lyme reflex	Positive, 2.78**
CMV serology (IgM)	<0.2
CMV serology (IgG)	>0.8**
Quantiferon-TB Gold Plus	Indeterminate (0.08-0.02)***

**Table 3:** Rheumatologic and hematologic workup ANA: anti-nuclear antibody; Ab: antibody; RNP: anti-nuclear ribonucleoprotein; Scl: scleroderma; ANCA: anti-nuclear cytoplasmic antibody; SPEP: serum protein electrophoresis; BM: bone marrow

Tests	Results
Rheumatoid factor	11.2
ANA titer	<1:40
Anti-JO1 Ab	<0.2
Anti RNP Ab	<0.2
Anti Scl-70 Ab	<0.2
Anti-Smith Ab	<0.2
Smooth muscle Ab	Negative
SS-A/SS-B Ab	<0.2/<0.2
ANCA	Negative
Direct-anti-globulin test	Negative
SPEP	No evidence of myeloma
Bone marrow biopsy	Negative for lymphoma, leukemia, with normocellular BM; maturing trilineage hematopoiesis

**Figure 1:**
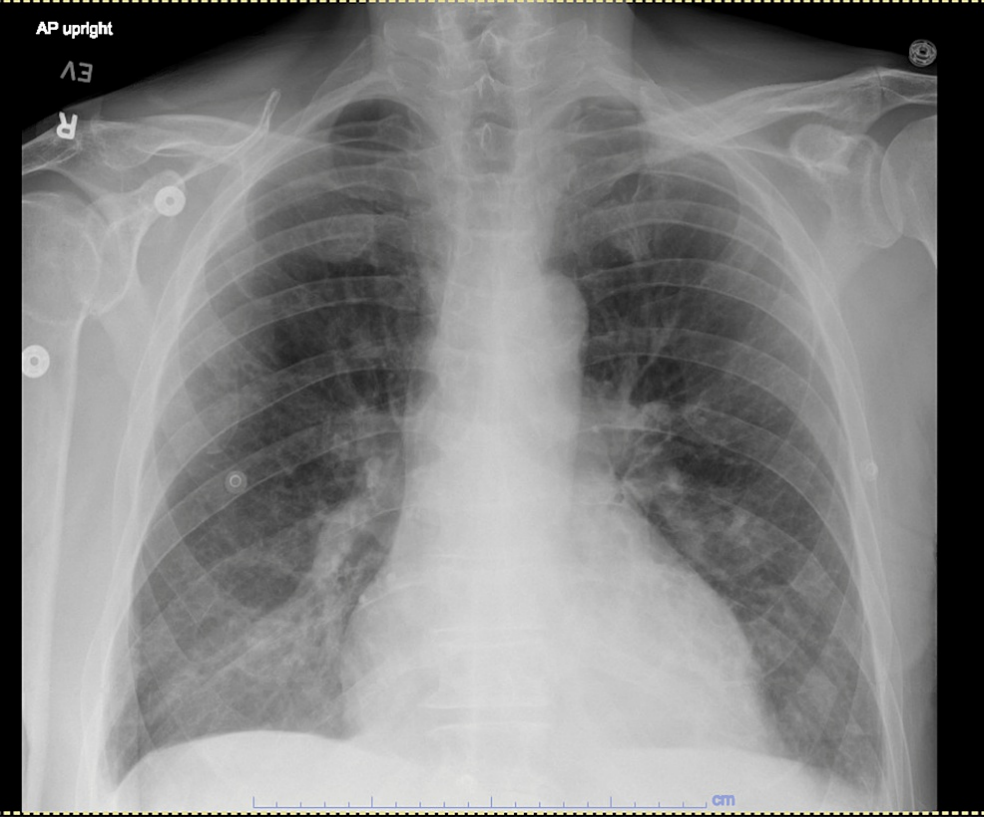
Chest X-ray Bilateral opacities were noted, most prominent on the right side

**Figure 2:**
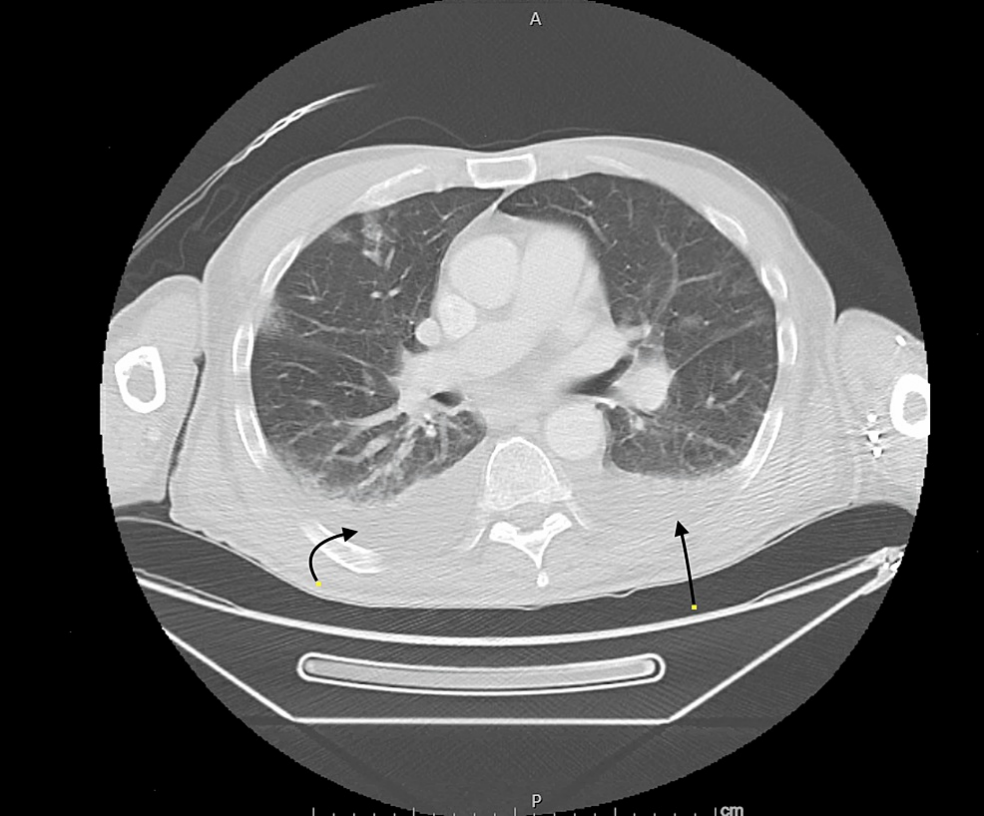
CT of the chest: axial view Bilateral pleural effusions indicated with black arrows (chronic) CT: computed tomography

**Figure 3:**
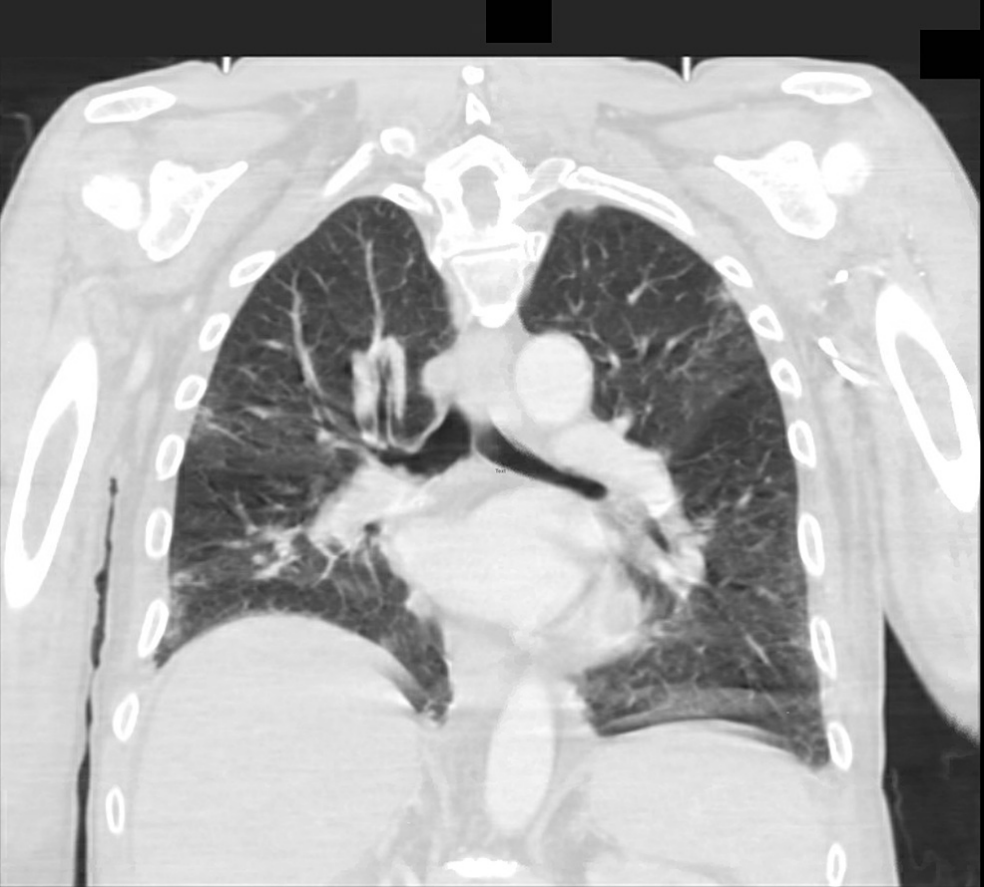
CT of the chest: coronal view CT: computed tomography

**Figure 4:**
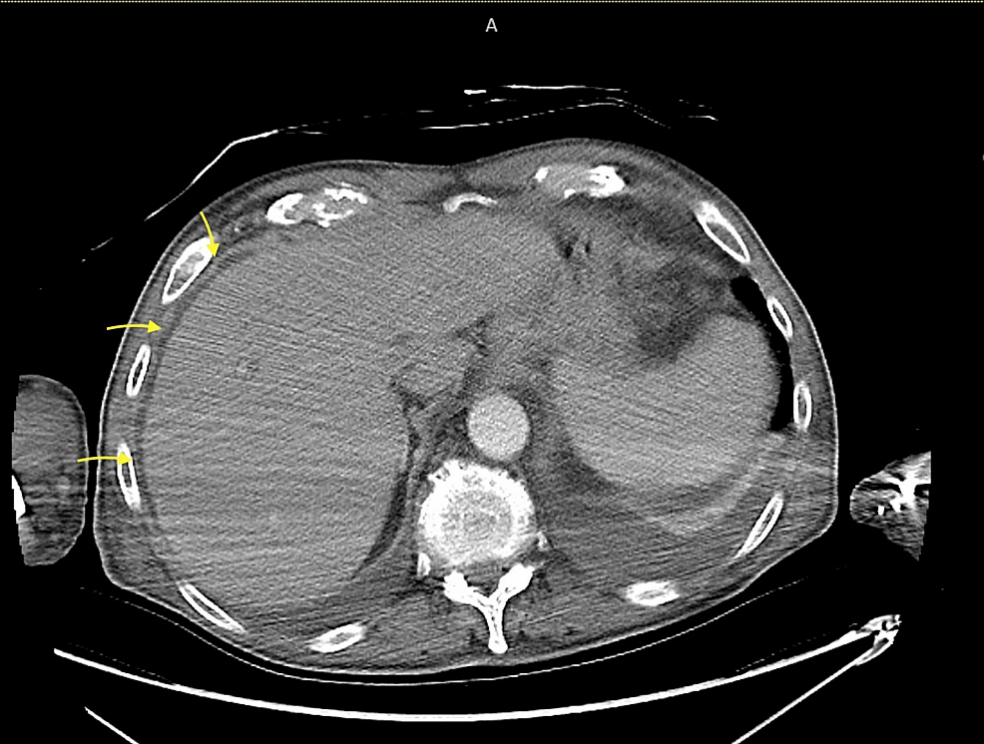
CT of the abdomen: axial view A small amount of peri-hepatic fluid is seen (indicated with yellow arrows) CT: computed tomography

Transthoracic echocardiogram, CT scan of the brain, chest X-ray (Figure 1), and CT scan of the chest (Figures 2, 3) and abdomen (Figure 4) were unremarkable.

Given persistent septic physiology, the patient received broad-spectrum antibiotics without a meaningful response. Malignancy and autoimmune pathophysiology were considered with the testing of antibodies (Table 3), which were largely non-diagnostic.

HLH vs. progressive Still’s disease was considered. After the initiation of dexamethasone, IVIg, and granulocyte-stimulating factors, his fever and mental status improved, although with recalcitrant shock, pancytopenia, and DIC. WBC count dropped to 400, and platelets to <10,000; D-dimer peaked at 17,500, and fibrinogen nadir of 28 mg/dL was observed. The soluble interleukin-2 receptor (sIL-2R or sCD25) was markedly elevated at 61,045 U/mL (reference range: 175.3-858.2 U/mL).

The patient was managed for suspected HLH with dexamethasone, IVIg, and granulocyte-stimulating factor. After a week of therapy, he had no significant clinical response. He had presented late into the course of his illness, and given the guarded prognosis, the family decided to transition him to comfort measures. He died the following day. 

The autopsy showed pulmonary congestion and diffuse alveolar damage with foci of interstitial chronic inflammation; the spleen was enlarged, weighing 250 g with preserved architecture and red pulp congestion, and adrenal glands showed diffuse lymphocytic and histiocytic infiltrate with positive CD3 and CD68 stains. Acid-fast bacilli (AFB), Grocott methenamine silver (GMS), and gram stains were negative.

## Discussion

Almost three years into the COVID-19 pandemic, its pathological sequelae are still under investigation. A myriad of inflammatory pathophysiology developing as a result of SARS-CoV-2 infection have been described [7,8,9,10]. This case suggests that unchallenged inflammation driven by COVID-19 may lead to HLH, with devastating outcomes in vulnerable patients. Early recognition and treatment of HLH are paramount for preventing progressive disease and mortality [11].

HLH refers to a hyper-inflammatory syndrome with phagocyte and cytokine-driven tissue damage without adequate immune regulation of macrophages and CD8+ lymphocytes. Hemophagocytosis of host blood cells by macrophages may lead to cytopenias and the ensuing cytokine storm from persistent inflammation can cause multi-organ failure [12]. While HLH is recognized as a distinct diagnosis, the pathophysiology falls on a spectrum of illnesses. Untreated, it is nearly always fatal, but with early and appropriate treatment, most patients survive. Therefore, sHLH is considered a hematological emergency, and early recognition is critical [11,13].

HLH should be considered in patients with compelling signs and symptoms with otherwise unexplained cytopenias and markers of inflammation, including elevated ferritin, sCD25, and CXCL9 [14]. The diagnosis is reached if at least five of the following nine criteria are met: fever, splenomegaly, peripheral blood cytopenias, elevated ferritin, hypertriglyceridemia or hypofibrinogenemia, elevated soluble CD25 or elevated CXCL9, demonstrable hemophagocytosis, or evidence of low or absent NK cell activity [13,15]. The HLH criteria and HScore can be used when clinical suspicion is high [15], and combined, these have a 93% sensitivity and 86% specificity [16,17].

Our patient’s initial symptoms of chronic fever, malaise, headaches, and arthralgia are nonspecific, but common in macrophage activation syndromes including HLH. After a medical evaluation, he met six of the criteria listed above: unexplained fever, splenomegaly, elevated ferritin, hypertriglyceridemia, hypofibrinogenemia, multiple cytopenias, and elevated sCD25. He did not show any evidence of hemophagocytosis on bone marrow biopsy, although this is not required for diagnosis. Based on HScore analysis, our patient scored 249 points, with a probability for sHLH of >99%.

## Conclusions

We hypothesize that HLH developed in our patient as an extreme outcome of a broader hyperinflammatory state, driven initially by his previous COVID-19 infection. Given the protracted symptomatic duration, we hypothesize that his initial SARS-CoV-2 infection caused a state of MIS, with subsequent macrophage activation and features of adult-onset Still’s disease. As he did not seek medical attention for months, it is difficult to apply the Yamaguchi diagnostic criteria for adult Still’s disease, although he did suffer from prolonged fever and arthralgia for months before presentation. We theorize that his macrophage activation progressed, leading to hemophagocytosis with multi-organ failure. Unfortunately, as his disease was in an advanced state at the initial presentation itself, the management turned out to be ineffective. Delayed diagnosis remains the greatest barrier to managing HLH. Treatment is most effective if provided early in the disease course, preferably at the onset of clinical suspicion itself.
